# Relationship of serum lipid parameters with kidney function decline accompanied by systemic arterial stiffness: a retrospective cohort study

**DOI:** 10.1093/ckj/sfad131

**Published:** 2023-05-26

**Authors:** Daiji Nagayama, Yasuhiro Watanabe, Takashi Yamaguchi, Kentaro Fujishiro, Kenji Suzuki, Kohji Shirai, Atsuhito Saiki

**Affiliations:** Department of Internal Medicine, Nagayama Clinic, Oyama, Tochigi, Japan; Center of Diabetes, Endocrinology and Metabolism, Toho University, Sakura Medical Center, Sakura, Chiba, Japan; Center of Diabetes, Endocrinology and Metabolism, Toho University, Sakura Medical Center, Sakura, Chiba, Japan; Center of Diabetes, Endocrinology and Metabolism, Toho University, Sakura Medical Center, Sakura, Chiba, Japan; Japan Health Promotion Foundation, Shibuya-Ku, Tokyo, Japan; Japan Health Promotion Foundation, Shibuya-Ku, Tokyo, Japan; Department of Internal Medicine, Mihama Hospital, Chiba, Chiba, Japan; Center of Diabetes, Endocrinology and Metabolism, Toho University, Sakura Medical Center, Sakura, Chiba, Japan

**Keywords:** arterial stiffness, cardio-ankle vascular index, kidney function decline, lipid parameters

## Abstract

**Background:**

Dyslipidemia is associated with kidney function decline (KFD), although the non-linear relationship of lipid parameters to KFD has not been fully elucidated. We aimed to determine the detailed relationship of baseline lipid parameters with KFD, considering the mediation of arterial stiffness.

**Methods:**

A total of 27 864 urban residents with estimated glomerular filtration rate (eGFR) ≥60 mL/min/1.73 m^2^ at baseline, who participated in a median of three (range two to eight) consecutive annual health examinations were studied. Arterial stiffness was assessed by cardio-ankle vascular index (CAVI). KFD was defined as development of eGFR <60 mL/min/1.73 m^2^.

**Results:**

During the study period, 1837 participants (6.6%) developed KFD. Receiver operating characteristic analysis determined that the cutoff values independently associated with KFD are 123 mg/dL for low-density lipoprotein cholesterol (LDL-C) [area under the curve (95% confidence interval) 0.570 (0.557–0.583)], 65 mg/dL for high-density lipoprotein cholesterol (HDL-C) [0.552 (0.539–0.566)], 82 mg/dL for triglycerides (TG) [0.606 (0.593–0.618)] and 1.28 for TG/HDL-C ratio [0.600 (0.587–0.612)]. These cut-offs were independently associated with KFD in Cox analysis. Regarding the contribution of each lipid parameter to KFD, a linear relationship was observed for both TG and TG/HDL-C, and a U-shaped relationship for HDL-C. A adjusted mediating effect of CAVI on the relationship of TG or TG/HDL-C ratio with KFD was observed (mediating rate: 2.9% in TG, 2.5% in TG/HDL-C ratio). Regarding the association to KFD, a linear relationship was observed for both TG and TG/HDL-C, and a U-shaped relationship for HDL-C. A mediating effect of CAVI on the relationship of TG or TG/HDL-C ratio with KFD was observed after adjustment for confounders.

**Conclusions:**

TG and TG/HDL-C ratio related linearly to KFD and this was partially mediated by CAVI. A U-shaped relationship was observed between HDL-C and KFD risk. LDL-C showed no significant association. Further study should investigate whether intensive TG-lowering treatment prevents KFD via decreasing CAVI.

## Graphical Abstract

**Graphical Abstract:**
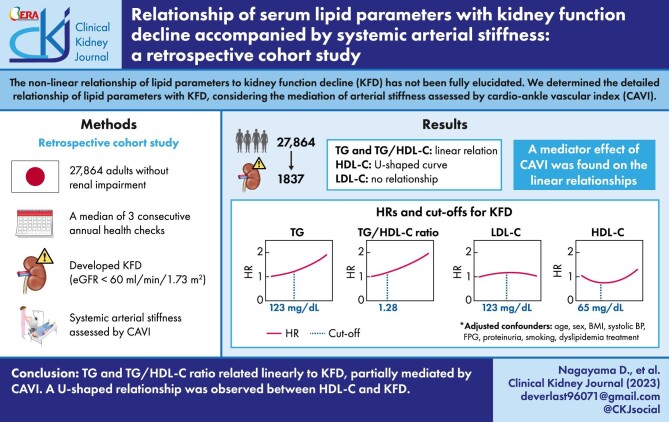

## INTRODUCTION

Chronic kidney disease (CKD) is one of the leading causes of morbidity and mortality in the 21st century [1]. Therefore, strategies have focused on the detection and treatment of CKD to prevent progression to end-stage kidney disease and various clinical outcomes caused by CKD [2]. Serum lipid abnormalities are known to be a risk factor for cardiovascular disease (CVD), and an association between lipid metabolism and kidney impairment has also been postulated. Moorhead *et al*. [3] first proposed the “lipid nephrotoxicity” hypothesis in 1982, suggesting that abnormalities in lipid metabolism precede CKD. Subsequently, a number of longitudinal studies have demonstrated that baseline lipid parameters predict future development of CKD [4–11]. A common finding of these reports is the assumption of an independent linear relationship between lipid parameters and the development of CKD. In contrast, when analyzing medical cohort studies, it is also important to consider non-linear models to accurately capture the relationship between explanatory and objective variables.

Abnormalities in vascular function, as assessed by systemic arterial stiffness, appear with aging, and are associated with several atherosclerotic diseases. Some CKD is caused by atherosclerosis, and CKD *per se* can lead to a vicious circle of accelerated atherosclerosis [12, 13]. Increased arterial stiffness reflects reduced vascular distensibility, and may be a potential therapeutic target for cardiometabolic complications. Pulse wave velocity (PWV) is recognized as the gold standard in the assessment of arterial stiffness because of its established association with CVD events [14]. However, the difficulties in calculating PWV are related to its intrinsic dependence on blood pressure (BP) and the heterogeneity of arterial stiffness along the arterial tree [15, 16]. This raise concerns that PWV may underestimate the extent of vascular dysfunction due to CVD risks other than hypertension. The cardio-ankle vascular index (CAVI) has therefore been established as an alternative index to PWV, because it is not BP-dependent and includes the whole arterial tree comprising the aorta, femoral artery and tibial artery. This parameter is originally derived from the stiffness parameter β proposed by Hayashi *et al*. [17] and Kawasaki *et al*. [18], and reflects both the severity of various CVD risks and the effect of appropriate treatment interventions [19]. Furthermore, CAVI has been reported to predict not only CVD events [20, 21] but also kidney function decline (KFD) more effectively than PWV in the general Japanese population [22]. On the other hand, despite the independent association of each lipid parameter with CAVI [23], it remains unclear whether the pathophysiology of lipid nephrotoxicity is kidney-specific or mediated by systemic arterial stiffening.

With this background, the present retrospective cohort study aimed to verify the linear or non-linear contribution of each lipid parameter to KFD and determine the cut-off values of serum lipid parameters for the prediction of KFD. Furthermore, the mediating effect of arterial stiffening assessed by CAVI on the relationship between the two was also investigated.

## MATERIALS AND METHODS

### Subjects and design

The population-based sample used in this retrospective cohort analysis consisted of 34 662 Japanese residents in major cities nationwide, who participated in the annual CVD and cancer screening program organized by the Japan Health Promotion Foundation, and had undergone two to eight consecutive annual health examinations between 2010 and 2018. The participants were unpaid volunteers who were not recruited for this study (unlike subjects of a clinical trial). Of the 34 662 subjects who were assessed for eligibility, those with insufficient data (*N* = 5062) and those with an estimated glomerular filtration rate (eGFR) <60 mL/min/1.73 m^2^, or a history of any kidney disease at the first examination (baseline) (*N* = 1736), were excluded. Finally, a total of 27 864 subjects with eGFR ≥60 mL/min/1.73 m^2^ at baseline were included in the study, and these subjects participated in a median of three (range two to seven) consecutive annual examinations.

#### Ethics approval and consent to participate

The protocol of the study was prepared in accordance with the Declaration of Helsinki, and this study was reviewed and approved by the Institutional Review Board and Ethics Committee of Sakura Hospital, School of Medicine, Toho University (No. S20091). Written informed consent for the examinations was obtained from the participants, and informed consent to participate in this study was obtained by opt-out method.

### Data collection

All parameters were assessed using standardized methods. Height and body weight (BW) were measured, and body mass index (BMI) was calculated as follows: BW (kg) divided by square of height (m). BP was measured from the upper arm cuffs in a sitting position after 5-min rest. Blood samples were collected from the antecubital vein in the morning after a 12-h fast to measure fasting plasma glucose (FPG, mg/dL), total cholesterol (TC, mg/dL), triglycerides (TG, mg/dL) and high-density lipoprotein cholesterol (HDL-C, mg/dL). Low-density lipoprotein cholesterol (LDL-C) (mg/dL) was calculated using Friedewald formula: LDL-C = (TC) ‒ (HDL-C) ‒ (TG/5). This formula is not valid for patients with TG ≥ 00 mg/dL [24]. Therefore, subjects with TG ≥400 mg/dL (*N* = 299, 1.07%) were excluded from the analysis of LDL-C only. The TG/HDL-C ratio, which has recently been reported to have predictive ability for KFD [7, 10], was calculated as TG level divided by HDL-C level. In addition, the non-traditional lipid parameters were calculated by following formulas:

Atherogenic index of plasma[25] = Log_10_ (TG/HDL-C)

Non-HDL cholesterol (Non-HDL-C) [26] = TC-HDL-C

Atherogenic coefficient [25] = Non-HDL-C/HDL-C ratio

Castelli risk index (CRI) -1 [25] = TC/HDL-C ratio

CRI-2 [25] = LDL-C/HDL-C ratio

Although there are several formulas for estimating GFR, a general practice and public health perspective favors the Chronic Kidney Disease Epidemiology Collaboration (CKD-EPI) equation [27]. This formula was devised in 2009 by the National Kidney Foundation using National Health and Nutrition Examination Survey (NHANES) 1999–2006 data. Meanwhile, the Japanese Society of Nephrology launched the “Japanese GFR Estimation Formula” project with the aim of estimating GFR with a method more suitable for the Japanese population than the conventional formula. Subsequently, Matsuo *et al*. developed a formula to estimate kidney function from Japanese data [28]. Against this background, the following Japanese-specific formula was adopted in the present analysis of large-scale cohort data from the Japanese population. The eGFR was calculated by the following equation from the Japanese Society of Nephrology:


\begin{eqnarray*}{\mathrm{eGFR}}( {{\mathrm{mL}}/\min /1.73\,{{\mathrm{m}}^2}} ) &=& 194 \times {\mathrm{creatinin}}{{\mathrm{e}}^{ - 1.094}}\\
&&\times {\mathrm{ag}}{{\mathrm{e}}^{ - 0.287}}\left( { \times 0.739\,\,{\mathrm{if\ female}}} \right).\end{eqnarray*}

KFD was defined as eGFR <60 mL/min/1.73 m^2^, corresponding to GFR category 3a or worse [29]. Since all the subjects had eGFR ≥60 mL/min/1.73 m^2^ at the first health examination, a decrease of eGFR to <60 mL/min/1.73 m^2^ at any subsequent annual health examination during the study period was defined as development of KFD.

Spot urine samples were collected and used for urinalysis by dipstick method. Urinalysis results were recorded as (–), (+/–), (1+), (2+) and (3+). Proteinuria was defined as urinary protein (1+) or above, which corresponds to a urine protein level of 30 mg/dL or higher.

Current smoking and habitual alcohol consumption status were determined by a questionnaire. Habitual alcohol consumption was defined as daily drinking.

### Measurement of arterial stiffness parameters and blood pressure

Arterial stiffness was assessed by CAVI using VaSera VS-1500 (Fukuda Denshi Co. Ltd, Tokyo, Japan). CAVI was calculated using the following formula [16]:

CAVI = a{2ρ × ln(Ps/Pd)/ΔP × PWV^2^} + b, where Ps is systolic BP (SBP); Pd is diastolic BP (DBP); ΔP is Ps – Pd; ρ is blood density; PWV denotes cardio-ankle PWV, and a and b are constants.

The cuffs were wrapped around the upper arms and ankles of a subject in spine position with the head in the midline position. The test was started after 5 min of rest. When detecting pulse waves in the upper arms and ankles with cuffs, a low cuff pressure of 30–50 mmHg was used to minimize the influence of cuff pressure on hemodynamics.

### Statistical analysis

The SPSS software (version 27.0.1, Chicago, IL, USA) or EZR (version 1.54, Saitama Medical Center, Jichi Medical University, Saitama, Japan) [30] was used for statistical analyses. All data are expressed as median (interquartile range) or percentage. Mann–Whitney *U* test or Fisher's exact test was performed to examine differences in baseline characteristics between individuals with and those without KFD. Using receiver operating characteristic (ROC) curve analysis combined with Youden's J index, the areas under the ROC curves [95% confidence interval (95% CI)] were calculated and the best discriminating levels of lipid parameters for KFD were determined. Covariate-adjusted event-free curves for KFD were generated from Cox proportional hazards models to estimate the differences in the time to KFD between groups. Cox proportional hazards analysis was also performed to identify the relationship with the variables to KFD, and the result is expressed as hazard ratio (HR) with 95% CI. Observation time was censored when an event (i.e. KFD) was identified. Participants without event were censored at the last contact until consecutive annual inspections (up to a maximum of seven) prior to 2018. Wald-test was conducted to assess the probability of the null hypothesis of no difference in all groups stratified by lipid parameter. Analysis of variance (ANOVA) was used to compare adjusted CAVI stratified by tertiles of lipid parameters. The F-value in ANOVA was calculated by dividing the mean square between groups by the mean square within groups. Mediation analysis was carried out using PROCESS (version 4.0) in SPSS [31]. The total effect must be significant to ensure the presence of mediation. Partial mediation exists when both indirect and direct effects are significant. The mediation rate (%) indicates the contribution of the mediation to the total effect and is calculated using the following formula: indirect effect/total effect ×100. Factors significantly differentiated by the presence or absence of KFD were adopted as covariates to adjust for HRs, CAVI or mediation effects. When FPG and SBP were adopted as covariates, treatment for diabetes and hypertension was not adopted due to their internal correlations, while treatment for dyslipidemia was adopted. In all comparisons, two-sided *P*-values <.05 were considered statistically significant. The difference between variables was also considered statistically significant if the 95% CIs did not overlap.

## RESULTS

### Comparison of baseline clinical and biochemical characteristics between with and without kidney function decline

Of the 27 864 participants (median age 46 years, male 44.4%), 1837 (6.6%) developed KFD during the study period between 2010 and 2018. Table 1 compares the baseline clinical characteristics of participants who did and those who did not develop KFD. Subjects with KFD were found to have significantly higher male ratio, age, BMI, BP, CAVI, FPG, TC, LDL-C, TG, TG/HDL-C ratio, Log_10_(TG/HDL-C), non-HDL-C (mg/dL), non-HDL-C/HDL-C ratio, TC/HDL-C ratio, LDL-C/HDL-C ratio, creatinine serum level, frequency of proteinuria, and frequency of current treatment for hypertension, diabetes or dyslipidemia; and significantly lower HDL-C and eGFR. In addition, frequency of current smoking tended to be lower in individuals with KFD (*P* = .061).

**Table 1:** Comparison of baseline clinical and biochemical characteristics in individuals with and those without KFD during the study period.

Variables	Individuals without KFD (*N* = 26 027)	Individuals with KFD (*N* = 1837)	*P*-value
Male gender (%)	44.2	47.1	.018
Age (years)	45 (36–56)	61 (52–68)	<.001
Height (m)	1.62 (1.56–1.70)	1.61 (1.55–1.68)	<.001
BMI (kg/m^2^)	21.9 (19.9–24.3)	22.9 (20.8–24.9)	<.001
SBP (mmHg)	116 (107–127)	126 (113–138)	<.001
DBP (mmHg)	72 (65–80)	78 (70–86)	<.001
CAVI	7.5 (6.9–8.2)	8.4 (7.7–9.1)	<0.001
FPG (mg/dL)	85 (80–91)	89 (84–97)	<.001
TC (mg/dL)	208 (185–235)	217 (195–241)	<.001
LDL-C (mg/dL)	121 (100–144)	130 (109–150)	<.001
HDL-C (mg/dL)	67 (56–81)	63 (53–77)	<.001
TG (mg/dL)	78 (55–117)	96 (69–139)	<.001
TG/HDL-C ratio	1.14 (0.73–1.97)	1.51 (0.96–2.43)	<.001
Log_10_(TG/HDL-C)	0.06 (–0.14 to 0.29)	0.18 (–0.02 to 0.39)	<.001
Non-HDL-C (mg/dL)	138 (114–165)	149 (127–174)	<.001
Non-HDL-C/HDL-C ratio	2.03 (1.51–2.75)	2.33 (1.77–3.09)	<.001
TC/HDL-C ratio	3.03 (2.51–3.75)	3.33 (2.77–4.09)	<.001
LDL-C/HDL-C ratio	1.78 (1.36–2.36)	2.03 (1.57–2.63)	<.001
Creatinine (mg/dL)	0.68 (0.59–0.81)	0.77 (0.69–0.92)	<.001
eGFR (mL/min/1.73 m^2^)	82.5 (74.6–92.0)	65.6 (62.6–69.6)	<.001
Proteinuria (%)	5.0	7.4	<.001
Current smoking (%)	35.1	32.9	.061
Habitual alcohol drinking (%)	34.2	33.1	.331
Receiving treatment for			
Hypertension (%)	5.4	22.8	<.001
Diabetes mellitus (%)	3.9	6.8	<.001
Dyslipidemia (%)	6.0	15.1	<.001

Data are presented as median (interquartile range) or percentage. Comparison of two groups was performed using Mann–Whitney U test for continuous variables and Fisher's exact test for dichotomous variables. KFD was defined as the development of eGFR <60 mL/min/1.73 m^2^ during the study period.

Based on these findings, the following confounders were used in subsequent analyses to calculate the contribution of lipid parameters to KFD: age, sex, BMI, SBP, FPG, proteinuria and frequency of current smoking.

### Cut-off values of lipid parameters and areas under receiver operating characteristic curves for kidney function decline

Next, the strength of the predictive power of each lipid parameter for KFD was quantified as shown in Table 2. ROC curve analysis together with Youden's J index identified the cut-off values of lipid parameters in predicting KFD as follows: 123 mg/dL for LDL-C [area under the curve (95% CI) 0.570 (0.557–0.583)], 66 mg/dL for HDL-C [0.552 (0.539–0.566)], 82 mg/dL for TG [0.606 (0.593–0.618)], 1.28 for TG/HDL-C ratio [0.600 (0.587–0.612)], 0.045 for Log_10_(TG/HDL-C) [0.600 (0.587–0.612)], 138 for non-HDL-C (mg/dL) [0.590 (0.588–0.603)], 2.03 for non-HDL-C/HDL-C ratio [0.591 (0.578–0.604)], 3.03 for TC/HDL-C ratio [0.591 (0.578–0.604)] and 1.604 for LDL-C/HDL-C ratio [0.585 (0.572–0.598)]. The predictive accuracies of TG and TG/HDL-C were greater than those of LDL-C and HDL-C. On the other hand, the ability of the non-traditional lipid parameters was not superior to that of TG and TG/HDL-C ratio, so they were excluded from this later analysis.

**Table 2:** Cut-off values of lipid parameters for predicting KFD obtained from ROC curve analysis.

Parameter	Cut-off	Sensitivity	Specificity	AUC (95% CI)	*P*-value
LDL-C	123	0.596	0.517	0.570 (0.557–0.583)	<.001
HDL-C	65	0.542	0.539	0.552 (0.539–0.566)	<.001
TG	82	0.634	0.531	0.606 (0.593–0.618)	<.001
TG/HDL-C ratio	1.28	0.595	0.557	0.600 (0.587–0.612)	<.001
Log_10_(TG/HDL-C)	0.045	0.676	0.485	0.600 (0.587–0.612)	<.001
Non-HDL-C	138	0.653	0.493	0.590 (0.588–0.603)	<.001
Non-HDL-C/HDL-C ratio	2.03	0.640	0.500	0.591 (0.578–0.604)	<.001
TC/HDL-C ratio	3.03	0.640	0.500	0.591 (0.578–0.604)	<.001
LDL-C/HDL-C ratio	1.604	0.734	0.399	0.585 (0.572–0.598)	<.001

Youden's J Index was used in conjunction with the ROC curve analysis to select the optimum cut-off points of lipid parameters for predicting KFD, defined as the development of eGFR <60 mL/min/1.73 m^2^ during the study period 10.1093/ckj/sfad131.

AUC, area under the ROC curve.

### Covariate-adjusted event-free curves of kidney function decline for subjects divided by cut-off value of each lipid parameter

**Figure 1:**
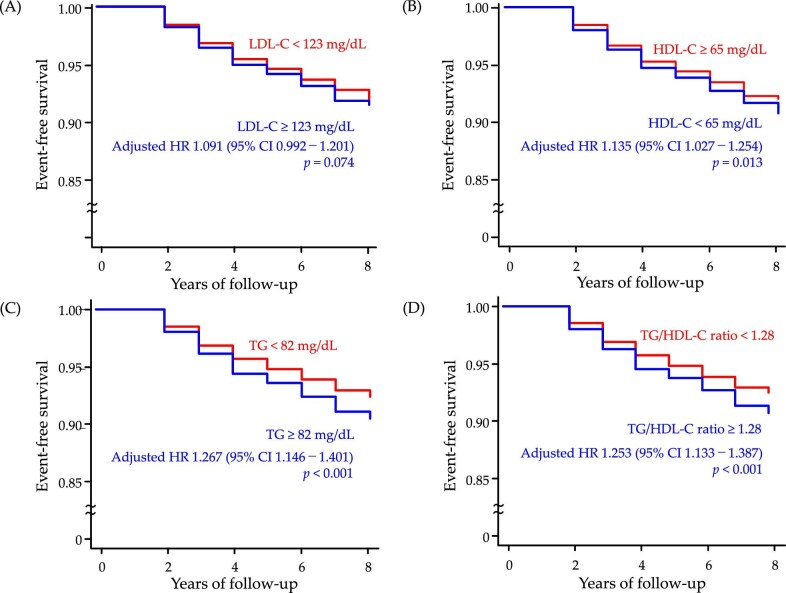
Covariate-adjusted event-free curves of KFD for two groups divided by the cut-off value of each lipid parameter. Participants were dichotomized into above and below the cut-off value of (**A**) LDL-C, (**B**) HDL-C, (**C**) TG and (**D**) TG/HDL-C ratio. Covariate-adjusted event-free curves estimated from Cox proportional hazards model were adjusted for age, sex, BMI, SBP, FPG, proteinuria, current smoking and dyslipidemia treatment.

Figure 1 shows the covariate-adjusted event-free curves of KFD comparing subjects with levels above and those with levels below the cut-off value of each lipid parameter, estimated by the multivariable Cox proportional hazards model. Participants were dichotomized according to whether their lipid levels were above or below the cut-off values shown in Table 2. The models were adjusted for confounders comprising age, sex, BMI, SBP, FPG, proteinuria and current smoking.

In this analysis, lower HDL-C (HDL-C <65 mg/dL) [adjusted HR (95% CI) 1.135 (1.027–1.254)], higher TG (TG ≥ 82 mg/dL) [1.267 (1.146–1.401)] and higher TG/HDL-C ratio (≥1.28) [1.253 (1.133–1.387)] independently related to KFD, while higher LDL-C (LDL-C ≥123 mg/dL) [1.091 (0.992–1.201)] did not.

### Relationship of adjusted hazard ratio for kidney function decline with each stratified lipid parameter

**Figure 2:**
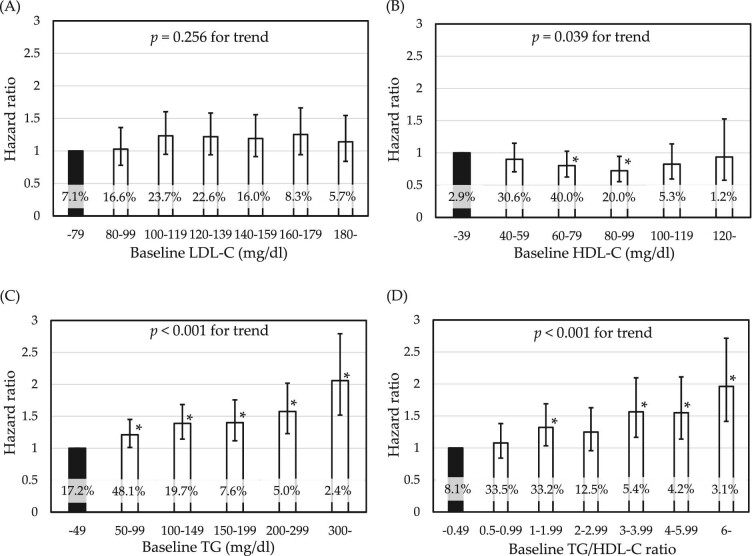
Relationship of adjusted HR for KFD with each stratified lipid parameter. HRs (95% confidence interval) for KFD were estimated using Cox proportional hazards analyses of stratified lipid parameters: (**A**) LDL-C, (**B**) HDL-C, (**C**) TG and (**D**) TG/HDL-C ratio. KFD was defined as the development of eGFR <60 mL/min/1.73 m^2^ during the study period. *Significant increase in HR versus the lowest stratum as the control group. *P*-values for trend were estimated using Wald-test. HRs were adjusted for age, sex, BMI, SBP, FPG, proteinuria, current smoking and dyslipidemia treatment. Percentage of individuals in each group is shown at the bottom of the bar.

Linearity or non-linearity of the contribution of each lipid parameter to KFD was examined by stratified analysis. Figure 2 shows the relationship of HR for KFD with strata of serum LDL-C level, HDL-C level, TG level or TG/HDL-C ratio. Wald-tests for trend showed that HR was significantly associated with HDL-C level, TG level or TG/HDL-C ratio, but not with LDL-C level. Furthermore, a U-shaped contributory relationship was found for HDL-C level; only subjects with HDL-C 60‒99 mg/dL showed lower HR for KFD compared with subjects with HDL-C <39 mg/dL. Increase in TG or TG/HDL-C ratio stratum appeared to be associated with a linear increase in HR for KFD.

### Adjusted CAVI stratified by tertile of each lipid parameter

**Figure 3:**
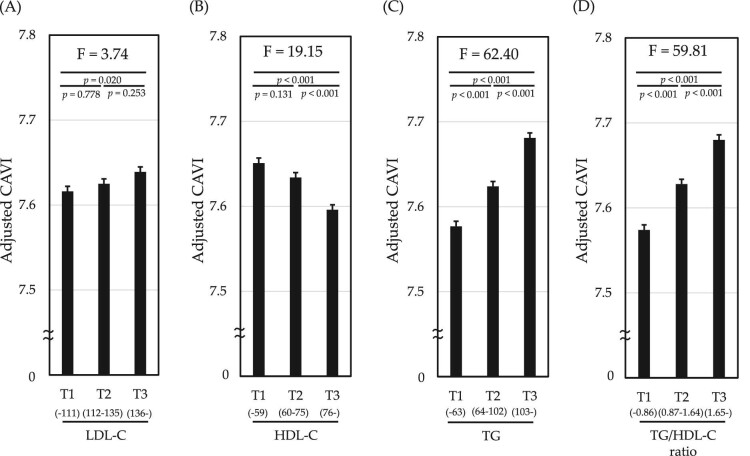
Comparison of adjusted CAVI stratified by tertile of each lipid parameter. Adjusted CAVI related to tertiles of lipid parameters: (**A**) LDL-C, (**B**) HDL-C, (**C**) TG and (**D**) TG/HDL-C ratio are shown. CAVI was adjusted by age, sex, BMI, SBP, FPG, proteinuria, current smoking and dyslipidemia treatment. Data are presented as mean ± standard error. *P*-values analyzed by ANOVA followed by *post hoc* Bonferroni method.

Additionally, we investigated the association of arterial stiffness with each lipid parameter stratified by tertile of serum level/ratio as shown in Fig. 3. Adjusted CAVI decreased with increasing tertile of HDL-C level (Fig. 3B) (mean T1:T2:T3 = 7.65:7.63:7.60), but increased with increasing tertiles of TG level (Fig. 3C) (7.58:7.62:7.68) and TG/HDL-C ratio (Fig. 3D) (7.57:7.63:7.68). In addition, trend tests yielded relatively large variances for TG (F = 62.40) and TG/HDL-C ratio (F = 59.81), compared with that for HDL-C (F = 19.15). On the other hand, no significant relationship between LDL-C level and CAVI was observed (Fig. 3A) [7.62:7.63:7.64].

### Mediation effect of CAVI on the relationship between lipid parameters and kidney function decline

**Figure 4:**
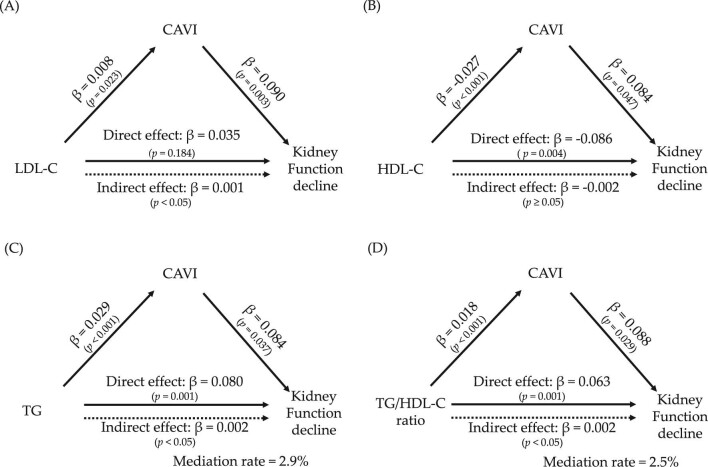
Mediated effect of CAVI on the associations between lipid parameters and KFD. Relations of (**A**) LDL-C, (**B**) HDL-C, (**C**) TG and (**D**) TG/HDL-C ratio with KFD are shown. Mediation analyses were adjusted for confounding factors of age, sex, BMI, SBP, FPG, proteinuria, current smoking and dyslipidemia treatment. KFD was defined as the development of eGFR <60 mL/min/1.73 m^2^ during the study period. β, standardized β coefficients.

Finally, as shown in Fig. 4, we examined whether the relationship between each lipid parameter and KFD was mediated by CAVI. In the mediation analysis, LDL-C showed no direct effect on KFD (Fig. 4A). The indirect effect of HDL-C on KFD was not significant (Fig. 4B). In contrast, for TG (Fig. 4C) and TG/HDL-C ratio (Fig. 4D), the partial mediation effect of CAVI on KFD remained significant even after adjustment for age, sex, BMI, SBP, FPG, proteinuria and current smoking (mediating rate: 2.9% in TG, 2.5% in TG/HDL-C ratio).

## DISCUSSION

In the present study, kidney function declined in 1837 participants (6.6%) during the study period. The cut-offs for HDL-C (65 mg/dL), TG (82 mg/dL) and TG/HDL-C ratio (1.28) obtained from ROC curve analysis were confirmed to relate independently to KFD. Regarding the adjusted contribution rates of each lipid parameter at stratified blood levels for KFD, a linear positive relationship was observed for both TG and TG/HDL-C, whereas a U-shaped relationship was observed for HDL-C. In other words, while HDL-C levels ranging from 40 to 99 mg/dL were associated with lower risk of KFD, levels above 100 mg/dL showed no such association. In contrast, LDL-C showed no significant trend. The mediating effect of vascular stiffness assessed by CAVI on the relationship of TG or TG/HDL-C ratio with KFD was confirmed even after adjustment for conventional risk factors. The novelty of this retrospective cohort study is as follows: first, confirmation of the U-shaped contribution of HDL-C to KFD; second, the finding that lipid nephrotoxicity associated with high TG and TG/HDL-C ratio is partially mediated by systemic arterial stiffening.

The cut-offs of HDL-C and TG for predicting KFD obtained in this study (HDL-C: 65 mg/dL, TG: 82 mg/dL) seem to be strict compared with the corresponding reference values (40 mg/dL and 150 mg/dL, respectively) for the prevention of CVD [32]. However, the present cut-off values are almost consistent with our previous report showing cut-offs of HDL-C 63 mg/dL and TG 93 mg/dL associated with high CAVI (≥9.0) [23], which are essentially the cut-offs for the presence of coronary artery stenosis [19, 33]. In addition, there is another rationale for tightening the normal range of serum TG level. Chan *et al*. [34] reported that men with visceral obesity and TG <150 mg/dL had higher levels of remnant-like particle cholesterol than lean subjects with TG <106 mg/dL. This finding would suggest that remnant cholesterol that exerts nephrotoxicity may appear even when TG level is within the conventional normal range [35]. Future studies are needed to confirm whether intensified treatment of hypertriglyceridemia is effective in preventing KFD.

Recently, the predictive ability of TG/HDL-C ratio for KFD has attracted attention [7, 10]. In the present study, the TG/HDL-C ratio showed almost the same predictive ability for KFD as TG alone. However, the relationship between HDL-C and KFD was U-shaped, which accords with the finding that hyper-HDL cholesterolemia had an adverse effect on CVD mortality in pooled analyses of cohorts [36–38]. With regard to these results, it has been suggested that severe hyper-HDL cholesterolemia may be atherogenic due to altered HDL particle shape and functional properties. Consequently, reduced accuracy of the TG/HDL-C ratio should be noted in individuals with HDL-C ≥100 mg/dL (6.8% of the all participants in this study). Nevertheless, as there were few individuals with HDL-C levels ≥100 mg/dL and TG/HDL-C ratios ≥1.28 in this study, it was not possible to compare the contribution of these high levels/ratios to KFD. Future verification is desirable.

Several studies have reported that elevated LDL-C may be associated with KFD [9, 11]. Conversely, it is also known that aggressive LDL-C-lowering therapy does not prevent KFD [39], and this finding is consistent with our results. Our results do not necessarily rule out nephrotoxicity due to LDL-C. However, lipid parameters related to insulin resistance may have stronger nephrotoxicity than LDL-C. In fact, increased LDL particles, intermediate-density lipoproteins and chylomicron remnants in the presence of high TG and TG/HDL-C ratio may relate to the development of CKD [7, 40]. These lipoproteins stimulate monocytes and macrophages to release inflammatory cytokines and chemokines via oxidative response and promote inflammation [41], resulting in KFD. Considering that CAVI can be improved by dyslipidemia treatment that reduces oxidative stress [42–44], the results of this study showing the association of lipid parameters except LDL-C with CAVI (Fig. 3) support the pathophysiological relationship between lipid parameters and CAVI. In addition, we have also reported that the antioxidant effect of probucol, an anti-hyperlipidemic drug, effectively prevents end-stage kidney disease [45]. These findings may suggest that, ultimately, we need to consider not only quantitative (levels), but also qualitative (oxidation) management for lipid disorder.

The evidence to date suggests a bidirectional causal relationship between changes in lipid metabolism and kidney function. It has been reported that CKD patients, especially those with nephrotic syndrome and CKD stage 5D, are more likely to develop secondary dyslipidemia such as hypertriglyceridemia and/or hypo-HDL cholesterolemia [46, 47]. The reason is that CKD induces delayed TG catabolism in the blood and the production of TG-rich lipoproteins in the liver through reduced activities of hepatic triglyceride lipase and lipoprotein lipase [48]. On the other hand, as shown in the present study, abnormalities in lipid metabolism may also promote KFD. In clinical practice, strict management of lipid parameters in addition to conventional standard CKD treatment may be necessary to break this vicious circle.

Limitations to this study are as follows. First, the cut-off of each lipid parameter independently associated with KFD in this study may only be applied to the middle-aged Japanese population. Next, we adopted the formula proposed by the Japanese Society of Nephrology to estimate eGFR [28], but it cannot be denied that this formula may deviate from the conventional Modification of Diet in Renal Disease/CKD-EPI equation. Furthermore, although creatinine serum level was adopted as an indicator of kidney function in this study, cystatin C is considered to be a more precise indicator. The fact that the dipstick method was used to determine urinary protein was also problematic because it was not precise. Since only 34% of the subjects in this study had HbA1c measured, we were forced to treat FPG as a representative glucose metabolism parameter. Finally, using a cohort that participates in regular physical examinations gives rise to the potential for selection bias. Individuals who participate in regular health screenings may be more health conscious and have a healthier lifestyle than those who do not participate. Therefore, caution should be exercised when generalizing the findings of such studies to the general population.

## CONCLUSIONS

TG and TG/HDL-C ratio related linearly to KFD and was partially mediated by CAVI. A U-shaped relationship was observed between HDL-C and KFD risk. LDL-C showed no significant association. Further study should verify whether intensive TG-lowering treatment prevents KFD via decreasing CAVI.

## Data Availability

The data that support the findings of this study are not publicly available because they contain information that could compromise the privacy of research participants. Further enquiries may be directed to the corresponding author.
